# Prevalence and Associations of Epiretinal Membranes in an Elderly English Population: The Bridlington Eye Assessment Project

**DOI:** 10.3390/jcm13030739

**Published:** 2024-01-27

**Authors:** Craig Wilde, Georgios D. Panos, Ali Pooschti, Hamish K. MacNab, Jonathan G. Hillman, Stephen A. Vernon, Winfried M. Amoaku

**Affiliations:** 1 Division of Ophthalmology and Visual Sciences, School of Medicine, Faculty of Medicine and Health Sciences, University of Nottingham, Nottingham NG7 2UH, UK; craig.wilde@nuh.nhs.uk (C.W.); ali.poostchi2@nuh.nhs.uk (A.P.); profsavernon@doctors.org.uk (S.A.V.); winfried.amoaku@nottingham.ac.uk (W.M.A.); 2 Department of Ophthalmology, Queen’s Medical Centre, Nottingham University Hospitals NHS Trust, Nottingham NG7 2UH, UK; 3 Medical Centre, Bridlington HU3 2JZ, UKjghillman@doctors.org.uk (J.G.H.)

**Keywords:** epiretinal membrane, ERM, prevalence, risk factors, ageing, hypertension, diabetes, stroke, English population, European population

## Abstract

**Purpose:** To determine the prevalence and risk factors of epiretinal membranes (ERMs) in an adult English population. **Methods:** The Bridlington Eye Assessment Project is a population-based study of eye disease among residents aged 65 years or older. Comprehensive interviews and ophthalmic examinations were conducted to assess potential risk factors. Digital mydriatic nonstereoscopic 30° colour fundus photography (CFP) was performed. ERMs were classified as primary/idiopathic or secondary on the basis of findings from the ocular examination and the structured questionnaire. Logistic regression models were used to determine the independence of potential risk factors for idiopathic ERMs. **Results:** In a comprehensive screening of 3588 patients aged over 65, we identified an eye-based prevalence of ERMs of 4.26% and a subject-based prevalence of ERMs of 6.88%. The majority of these cases were idiopathic in nature (90.7%), while 9.3% were secondary ERMs; predominantly, there was a history of cataract surgery (43.5%). No significant correlation between idiopathic ERMs and factors such as age, gender, diabetes, hypertension, a history of stroke, or the presence of AMD was found. **Conclusions:** The prevalence of ERMs in an elderly English population and the proportion of idiopathic and secondary ERMs are similar to previous reports. However, in elderly patients aged over 65 years, age is not a risk factor for the presence of idiopathic ERMs. The presence of diabetes, hypertension, a history of stroke, and AMD of any grade was not associated with ERMs.

## 1. Introduction

Epiretinal membranes (ERMs) are a common retinal condition among the elderly. Although mostly asymptomatic, they can lead to a significant loss of visual acuity, cause visual symptoms such as distortion and metamorphopsia, and negatively impact the quality of life [1,2,3]. They may occur in the absence of any comorbid ocular pathology other than posterior vitreous detachment (PVD), where they are termed idiopathic or primary ERMs [4]. Secondary ERMs are those associated with other ocular diseases, including previous intraocular surgery, various retinal pathologies including retinal breaks and detachments, retinal vein occlusions, diabetic retinopathy, retinitis pigmentosa, previous retinal laser/cryopexy, and uveitis [5].

The Beaver Dam Eye Study (BDES) was the first population-based report of ERM prevalence in 1994 [6]. Since then, many other large population studies have reported the epidemiology of ERMs, mostly utilising colour fundus photography (CFP); however, the more recent studies have additionally used spectral domain optical coherence tomography (SDOCT) [7,8]. These studies, across different ethnicities and geographical regions, demonstrate great heterogeneity in ERM prevalence, including 1% in a Chinese population and 18.5% among Latinos [9,10]. The reasons for such variation are likely complex and may relate to the study design, population demographics such as the age range, as well as the grading methodology and imaging techniques. A recent systematic review and meta-analysis of population-based studies concluded that in specific regions, including Europe, robust evidence for prevalence and risk factors for ERMs remains absent, with most studies included being carried out in the Pacific Rim nations of the USA, Australia, Japan, Singapore, and China [11]. Although it could be argued that many of these already include large Caucasian populations of European ancestry, it is possible that true ethnic and population variations exist and relate to genetic predispositions, lifestyle factors including smoking, or clinical comorbidities such as diabetes, which vary between countries [6,12]. Interestingly, in the Melbourne Collaborative Cohort Study, among an older Australian population, the prevalence of ERMs was almost twice as high in participants of Southern European origin compared to those of Northern European ancestry [13]. It could be argued that Southern Europeans have a more pigmented fundus when compared to their Northern European counterparts, and ERMs are subsequently more easily detectable. However, the difference in pigmentation does not seem adequate to explain the result. While some other studies report higher prevalence rates among subjects with more pigmented skin, such as the Singapore Indian Eye Study (SINDI) (10.5%) and the Los Angeles Latino Eye Study (LALES) (18.5%), others have reported lower prevalence measures than the largest Caucasian studies [14,15]. Examples include the Beijing Eye Study in China (2.2%) and the Funagata study in Japan (5.44%) [16,17]. On the other hand, a recent study on vitreoretinal interface changes in Ghanaian Africans reported an ERM prevalence of 13.2% (eye-based) [18].

To date, there are two European studies that report ERM prevalence among European populations. However, the UK Biobank study reports prevalence among a 25% subsample of subjects, all of whom have visual impairment, and is not representative of the general UK population [19]. The Maastricht Study in the Netherlands did not recruit patients randomly or consecutively from within their population. Subjects were recruited following media campaigns for volunteers and from various registries, including a regional diabetes patient registry. The study stratified recruitment according to known type 2 diabetes mellitus, with an oversampling of individuals with diabetes [20]. 

To address the paucity of data among European populations, we aimed to describe the prevalence of ERMs and associated risk factors in an adult English population using data from the Bridlington Eye Assessment Project (BEAP).

## 2. Materials and Methods

### 2.1. Study Population

The BEAP is a single-centre population-based study in East Yorkshire, England, aimed at assessing the efficacy of eye disease screening in a population aged 65 and over. The approach utilised clinical assessments conducted by trained optometrists alongside digital imaging technology. The principal ocular conditions investigated were age-related macular degeneration (AMD), cataracts, and glaucoma. An exhaustive description of the study’s design and methodology has been detailed in previous publications [21,22]. In summary, all permanent residents aged ≥65 years registered with the town’s only GP practice were invited to attend by letter on a street-by-street basis. Patients registered as blind, bed-bound, or known to have significant dementia were excluded from the study. The Scarborough and North Yorkshire Local Ethics Research Committee approved the study protocol (Ref No. PB/RH/02/288), and the study was conducted according to the recommendations of the Declaration of Helsinki. Study recruitment occurred between 5 November 2002 and 29 March 2006.

### 2.2. Interview and Examination Procedures

A trained research nurse conducted in-person interviews with all participants, employing a structured questionnaire to gather demographic details and information pertaining to their ophthalmic and medical histories, including diabetes, previous strokes, and hypertension. Specific histories of previous ophthalmic surgeries, diabetic retinopathy, glaucoma, and macular degeneration were sought. 

LogMAR VA was recorded for each eye corrected with both current glasses or contact lenses and a pinhole (Baylie Lovie no. 4 chart). A full biomicroscopic ophthalmic examination was then performed, including grading of lens status (LOCS III), intraocular pressure, central corneal thickness, and the presence of pseudophakia, which were documented during the slit lamp examination. A dilated fundus examination was performed using a 90D lens (Volk Optical, Mentor, OH, USA) by one of four specially trained optometrists. The findings were documented on a structured proforma by research staff, who maintained anonymity in the data recording process. A digital nonstereoscopic CFP was conducted using a fundus camera (model TRC NWS, Topcon, Tokyo, Japan) equipped with a 10-megapixel camera back (Nikon, Tokyo, Japan). For each eye, a single 30-degree field focused on the macula (corresponding to the Early Treatment for Diabetic Retinopathy Study standard field 2) was captured. In cases where the initial images were deemed unsatisfactory, they were promptly retaken.

### 2.3. Grading of Retinal Photographs

The methodology for grading macular pathology, specifically AMD, and the quality control and adjudication procedures, including assessments of intergrader reliability, have been detailed in a previous publication [23]. All fundus photographs were assessed in a masked fashion by a single ophthalmologist who had received training in image grading using standard definitions and grids as described by the International Classification System for AMD [24]. No medical records or subject demographic data were available during the grading process. Images were graded for macular ERMs using a grid with an outer diameter of 3000 µm, which was placed over the image during grading. No enhancement tools were used for the grading of ERMs. Epiretinal membranes were identified in keeping with the original definitions as used in the BDES [7] and were recorded as present if there was a patch or patches of irregular increased reflection from the inner retinal surface giving a ‘glinting, water-silk, and shifting light reflex’ without retinal folds or the presence of a more opaque and grey appearance on the inner retinal surface with superficial retinal folds or traction lines. ERMs outside the grid were graded as absent. 

### 2.4. Classification of ERM

ERMs were classified as primary/idiopathic or secondary on the basis of findings from the ocular examination and the structured questionnaire after data merging. Primary ERMs were recorded if no cause for ERM development was evident. Secondary ERMs were defined as those occurring in the eyes in the presence of ocular comorbidities, including previous retinal detachment or retinal tear, previous retinal laser or cryopexy, retinal vascular disease, previous cataract surgery, and diabetic retinopathy [6,25].

### 2.5. Definitions

In the context of this study, the diagnosis of diabetes mellitus was based on either a self-reported history of diagnosis by a physician or the receipt of drug treatment, including insulin or oral hypoglycaemic agents. Hypertension was identified through a self-reported history of diagnosis by a physician, the use of medication for hypertension, or the presence of elevated blood pressure observed during clinical measurements (systolic blood pressure ≥ 140 mmHg or diastolic blood pressure ≥ 90 mmHg).

### 2.6. Statistical Analysis

Data obtained from the grading process were inputted and subjected to internal quality control checks, with any identified discrepancies subsequently corrected. Statistical analysis was performed using MedCalc Statistical Software version 18.2.1 (MedCalc Software bv, Ostend, Belgium) and Jamovi version 2.4 (The Jamovi Project (2023), https://www.jamovi.org accessed on 15 December 2023). The prevalence of ERMs was determined by calculating the percentage of the total population in which cases were identified in either one or both eyes. For idiopathic ERMs, logistic regression models were used to determine the independence of potential risk factors. Potential associations included age, gender (female), history of stroke (yes or no), diabetes (present or absent), and hypertension (present or absent). Odds ratios (OR) and 95% confidence intervals (CI) were calculated. *p*-values less than 0.05 were deemed to indicate statistical significance. The goodness of fit of the logistic regression model was evaluated using the Hosmer–Lemeshow test. Additionally, a logistic mixed model, accounting for within-subject correlation, was used to determine the association between AMD and ERMs.

## 3. Results

In total, 3588 patients aged over 65 years old were screened, of whom 2017 were female (56.2% female and 43.8% male). The mean age ± SD of the patients was 74.3 ± 6.6 years. ERMs were detected in CFP in 306 eyes (eye-based prevalence of 4.26%) from 247 subjects (subject-based prevalence of 6.88%). Idiopathic ERMs were found in 224 patients (90.7%) and secondary ERMs in 23 patients (9.3%). A summary of the results is depicted in Table 1. 

The patients diagnosed with ERMs had a mean age of 75.1 ± 5.25 years. The BCVA for these patients, calculated on an eye-based analysis, was recorded at 0.26 logMAR ± 0.57. Regarding the gender distribution among ERM patients, there were 127 females and 120 males. In terms of associated health conditions, 22 of the ERM patients were identified as having diabetes. A significant number of patients, 122 in total, were found to have hypertension, and 19 patients had a history of stroke. Additionally, our data showed that among the ERM patients, 49 had dry AMD, while 2 patients were identified with wet AMD.

Table 2 illustrates a comprehensive summary of the characteristics of patients with ERMs.

Logistic regression analysis failed to show a correlation between the presence of idiopathic ERMs and factors such as age, gender (female), diabetes, hypertension, and a history of stroke (see Table 3). Among the 23 cases (9.3%) with secondary ERMs, the most frequent cause was a history of cataract surgery, accounting for 43.5% (10 out of 23). The model was a good fit for the data (Hosmer–Lemeshow test, *p* = 0.77). Moreover, there was no evidence of an association between ERMs and any AMD grade (all *p* > 0.05, Table 4).

## 4. Discussion

Our investigation into the prevalence and risk factors associated with ERMs in an elderly population offers a comprehensive global perspective, enriched by comparisons with findings from a diverse array of international studies. Our study, revealing an eye-based prevalence of 4.26% and a subject-based prevalence of 6.88%, presents a striking contrast to other studies. Notably, the Handan Eye Study reported a prevalence of 3.4% in a rural Chinese cohort, markedly lower than rates observed in other Asian populations and Caucasian groups [26]. This disparity in prevalence rates across different ethnicities, as also seen in the LALES, Melbourne Collaborative Cohort Study (MCCS), SINDI, and Singapore Malay Eye Study (SiMES), underscores the possible significant influence of ethnic and racial factors on ERM prevalence [13,14,15,27]. Contrary to findings from other studies where age emerged as a predominant risk factor, our study did not identify age as a significant factor, likely due to the exclusive focus on an elderly cohort over 65 years old. Previous research has indicated that the prevalence of ERMs increases with age until 75 years [6]. Given that the mean age in our study was 74.3 years, this aligns with the notion that beyond this age threshold, age may not be a primary risk factor. Furthermore, this finding is similar to the absence of age correlation in the recent report from Ghana [18]. Additionally, it is also plausible that the presence of cataracts can obscure the detection of very mild ERMs when using direct ophthalmoscopy or fundus photography. This oversight might contribute, at least in part, to underestimating the presence of ERMs in older patients, thereby potentially influencing the perceived lack of correlation between age and ERM risk. This also suggests that, within the older age group, other factors may play a more crucial role in the development of ERMs. Moreover, unlike several other studies, female gender was not identified as a risk factor in our study. This could be attributed to the specific demographic and health characteristics of our elderly cohort, where gender-related differences in ocular health might be less pronounced or overshadowed by age-related changes and comorbidities. This absence of gender correlation with vitreomacular interface changes is similar to that in the recent report from Ghana [18]. Furthermore, our findings indicate that the presence of diabetes, hypertension, and a history of stroke were not associated with ERMs. These results are particularly noteworthy as they contrast with common assumptions about these systemic conditions as risk factors for ocular diseases. Our study also found that AMD of any grade was not correlated with ERMs, a finding that aligns with the results of the Ghana AMD Study Group [18]. This consistency across different studies and populations suggests a more complex relationship between AMD and vitreoretinal interface changes than previously understood [28,29,30], potentially influenced by factors other than those commonly associated with AMD.

While increasing age was a consistent risk factor in most studies, the Handan Eye Study’s association of myopia with primary ERMs aligns with findings from the Visual Impairment Project (VIP) Study [26,31]. Intriguingly, an inverse association between current smoking and primary ERMs was observed in the Handan Eye Study, paralleling reports from the VIP and SiMES [26,27,31]. This could suggest a protective effect of smoking or a survival bias among smokers, a hypothesis warranting further exploration. The methodological choices, particularly the use of OCT versus CFP, significantly impact ERM detection and prevalence estimation. The Handan Eye Study’s integration of OCT with retinal photographic diagnosis likely reduced the underestimation of ERM prevalence, highlighting the critical role of diagnostic methodologies in epidemiological research [26]. The SiMES and SINDI studies, focusing on Malay and Indian populations in Singapore, reported higher ERM prevalence rates compared to Caucasian populations, challenging previous assumptions about lower ERM prevalence in Asian groups [14,27]. These findings, along with the higher prevalence observed in the LALES among Latinos, suggest a complex interplay of genetic, environmental, and lifestyle factors influencing ERM development across different ethnicities [15]. Our study, along with international comparisons, underscores the need for heightened clinical awareness and targeted screening strategies for ERMs, particularly in ageing populations. The varying prevalence rates and risk factor associations across different ethnicities and regions highlight the importance of culturally tailored public health interventions and eye care services. While our study and international comparisons provide valuable insights, they also reveal limitations, such as potential residual confounding and variations in diagnostic criteria and methodologies. Notably, our study’s lack of OCT examinations is a significant limitation. Although CFP, our employed method, is a gold-standard technique and our study’s results are comparable to most previous studies, the inclusion of OCT would have provided much higher sensitivity, crucial for detecting subtle retinal changes. Additionally, the absence of refractive error and axial length measurements in our study represents another limitation. These measurements are important for understanding the development and progression of ocular conditions, and their omission could impact the comprehensiveness of our findings.

Future research should focus on longitudinal studies to elucidate causal relationships, explore any possible genetic underpinnings of ERMs, and assess the impact of lifestyle factors such as diet and smoking. Additionally, advancements in imaging technologies (including OCT) would refine the detection and classification of ERMs, enhancing our understanding of their epidemiology and pathophysiology. A detailed future study exploring the longitudinal relationship between the vitreoretinal interface and the development of a posterior vitreous detachment and association with ERMs would be invaluable. Localised anatomical considerations, such as axial length and refractive status variations, would be useful future considerations in population studies.

In conclusion, our study, enriched by global comparisons, contributes significantly to the existing literature on ERM prevalence, highlighting the importance of methodological consistency, demographic considerations, and the need for ongoing research to elucidate the incidence and risk factors of ERMs in diverse populations and ethnic groups with a particular emphasis on age-specific cohorts and the nuanced role of gender.

## Figures and Tables

**Table 1:** A summary of the results of the study.

Total number of patients	3588
Age (mean ± SD)	74.3 ± 6.6 years
Gender distribution	2016 (56.2%) female, 1572 (43.8%) male
Eye-based ERM prevalence	4.26%
Subject-based ERM prevalence	6.88%
Idiopathic ERM	224 (90.7%)
Secondary ERM	23 (9.3%)

**Table 2:** A summary of the ERM patients’ characteristics.

Characteristic	Data
Mean age (years)	75.1 ± 5.25
Mean best corrected visual acuity (logMAR)	0.26 ± 0.57
Gender distribution	
*-Female*	127 (51.4%)
*-Male*	120 (48.6%)
Patients with diabetes	22 (8.9%)
Patients with hypertension	122 (49.4%)
Patients with stroke	19 (7.7%)
Patients with dry AMD	49 (19.8%)
Patients with wet AMD	2 (0.8%)

**Table 3:** Results of the logistic regression analysis.

Factor	Coefficient	Std. Error	Wald	Odds Ratio	95% CI	*p*
Age	0.013612	0.010315	1.7415	1.0137	0.9934 to 1.0344	*0.1869*
Gender (female)	−0.22238	0.13274	2.8064	0.8006	0.6172 to 1.0385	*0.0939*
Diabetes	−0.12292	0.23350	0.2772	0.8843	0.5596 to 1.3976	*0.5986*
Hypertension	−0.084716	0.13579	0.3892	0.9188	0.7041 to 1.1989	*0.5327*
Stroke	−0.20336	0.24978	0.6629	0.8160	0.5001 to 1.3314	*0.4155*

**Table 4:** Relationship between ERMs and AMD (logistic mixed model).

	Odds Ratio	95% CI	*p*
Dry AMD	1.80	0.0207 to 157	*0.797*
Geographic atrophy	0	-	*1.0*
Neovascular AMD	6.00 × 10^−10^	2.83 × 10^−226^ to 1.28 × 10^207^	*0.978*

## Data Availability

The data presented in this study are available on request from the corresponding author.
